# Theory of Sensation

**Published:** 1811-12

**Authors:** T. Smith

**Affiliations:** Bristol


### Medical and Physical Journal.

VOL. XXVI.] DECEMBER, 1811. [NO. 154.

Printed for R. PHILLIPS, by E. Hemsted, Great New Street, Fetter Lane, London.

*For the Medical and Physical Journal.*

### *Theory of Sensation.*

(Continued from P. 371.)

IN the small-pox, scarlatina, &c. the application of cold water to the surface of the body has been often practised with striking advantage. The rules which Dr. Currie has laid down with so much justness and precision, if strictly attended to, would, I firmly believe, render the use of this remedy in these diseases at once perfectly safe and highly salutary. It cannot however be altogether void of utility to ascertain, if possible, upon what principle it is that the good effects of cold applications to the skin in these diseases, have uniformly appeared to be confined to those cases in which it was agreeable, at least not disagreeable to the patient's sensations. Was this owing to its having *diminished* or *increased* action? This question cannot be decided without having particular cases in view: and I cannot do better than solicit the reader's attention to three cases of scarlatina, communicated by Dr. Gregory, Professor of the Practice of Medicine in the University of Edinburgh, to Dr. Currie, and published by him in the 2d Vol. of his Medical Reports. We may safely reason from these cases, as they contain a minute and faithful picture of the effects of cold ablution in scarlatina; by a physician whom it is impossible to suspect of any undue bias in favour of the practice. How much the cold and tepid ablutions relieved painful or uneasy sensation in these cases may be inferred from the following extract, from the end of the second letter: "I have had much pleasure," Dr. Gregory remarks, "in observing repeatedly in the youngest child

(the two-year-old gentleman) the great and immediate good effects of the cold or tepid washing, not only in lessening the frequency of pulse and heat of skin, but in relieving the febrile oppression and uneasiness. The little patient who just before was crying very much, unable to hold up its head, incapable of being pleased or amused with any thing, nay almost incapable of looking at any thing, immediately after being washed (I mean in two or three minutes) would begin to look up, and take notice of the people near him, then amuse himself with his play-things, then get upon his legs and run about upon the floor, and at last go quietly to sleep." Perhaps it may be urged that these striking effects arose from the morbid actions being diminished by the ablutions. But is it certain that any action essentially belonging to the disease was diminished? Were the actions by which the eruption is formed on the skin diminished? No, for the rash was permanent, and went its due course; nay, if I may judge from my own experience, the course of these actions is quickened by subduing uneasy sensations, whether that be done by bleeding, purgatives, or by the cold or tepid washing. Were the actions by which caloric is evolved diminished? A common effect of the cold washing (particularly when the benefit resulting from it is to be permanent) is, that a general, free and lasting perspiration succeeds to it.—Sweating is now known to be a very cooling process, under which the system could not possibly long maintain its temperature at the natural standard, much less above it, if the evolution of caloric were not considerably greater than natural. Hence it appears probable that the coolness of the body in the perspiration that succeeds to the cold affusion or washing, is not owing to a decrease of the actions by which caloric is evolved, but to an increase of the actions by which the body is cooled. But this is not all. When the cold ablution is not succeeded by sweating, or when the flow of sweat is not lasting, the temperature of the body rises, (as I have often observed, and as may be inferred from the cases on record), with *increased rapidity;* a fact which proves that the actions by which caloric is evolved are not only not diminished, but considerably increased by the abstraction of the redundant caloric. But the same increased evolution of caloric may be inferred to take place when the temperature of the system is kept down by copious sweating, which is only another way for removing the excessive caloric. In every point of view therefore it appears that the cold affusion, or ablution, in relieving painful or uneasy sensation, increases *action;* and that instead of diminishing the peculiar actions *already excited* by the contagious matter, it allows them to be performed with greater quickness and facility, facility, while at the same time it obviates the destructive effects of excessive temperature upon the actions and organization of the body.

There is perhaps no disease in which the prejudice against the use of cold applications has been more strongly and generally rooted than in *Erysipelas*. Cooling lotions, however, have been employed in this disease with advantage. Dr. Irvine strongly recommends them*. In a very able review of his work, when noticing this practice, the Reviewer makes the following observations: "† This practice is also commended by other writers ‡; and for our own parts, we have been in the constant habit of employing cooling lotions, such as equal parts of aq. ammonia acetatæ and water, &c. in erysipelatous inflammation, with great benefit; and with immediate relief to the feelings of the patient, which the application of farinaceous powders most commonly aggravated or failed to relieve; but very able surgeons deprecate the indiscriminate use of them" ||.

The *indiscriminate* use of cold applications in any disease, whether fever, gout, scarlatina, small-pox, or erysipelas, cannot be too much decried. The following case occurred in my own practice, and first taught me the necessity of attending to the sensations of the patient in the use of that remedy. A young woman was in the fourth day of typhus. Her skin was hot, her face flushed; she had violent headach, and was at intervals delirious. At a time when the delirium was urgent, I applied cloths wet in cold water and vinegar to her head, with the immediate effect of removing the delirium. The patient expressed great satisfaction, and said it relieved the headach. After renewing the cloths several times, the patient began to complain that she felt them too cold, and that they brought back the headach. At that time I had neither the rules which Dr. Currie has laid down, nor any other safe principle to direct me when to stop. Pleased, however, with the obvious good effects derived from the cold applications in the first instance, I encouraged my patient to bear them to be continued; thinking, perhaps, they would be as effectual in preventing the recurrence of the headach and delirium, as they had been in removing them. She submitted; but in a short time her skin became deadly cold; she was seized with

* See Observations on the Diseases of Sicily, Chap. 12.

† Edin. Med. and Surg. Journ. for July, 1811.

‡ See Cooper's Dictionary of Practical Surgery. See also his First Lines of the Practice of Surgery.

|| See Pearson's Principles of Surgery, Ch. 10, Sect. 4.

a low muttering delirium; and her countenance assumed a cadaverous appearance. Alarmed at these very dangerous symptoms, I had immediate recourse to warm applications, and succeeded in restoring a due warmth to the skin, and removing the delirium. After this she soon fell into a quiet sleep, which continued for some hours with a warm moisture of the skin, and awoke, much to my satisfaction, greatly relieved of all the febrile symptoms. There can be little doubt that if the cold applications had been longer continued in this case, the effects would have quickly proved fatal. To caution against the indiscriminate use of cold affusion in fever, Dr. Currie has very properly given a case which occurred in his practice, in which the cold affusion was used in the cold stage of a tertian, with very dangerous and alarming effects*. Elsewhere† he mentions, with just indignation, his having heard of two cases of scarlatina maligna, in which, on the supposed authority of the Medical Reports, several gallons of perfectly cold water were poured over the patients, at a time when they were under low delirium, with the *skin cool and moist and the pulse scarcely perceptible.* In a case of confluent small-pox, *in which the patient was constantly complaining of cold*, I have been informed, on good authority, that the medical attendant caused him to be taken out of bed, carried into the open air, and exposed for a considerable time, almost naked, to the wind in a bleak day in December. The patient, a boy about seven or eight years of age, soon became comatose and died the same day.

On the whole, I hope it will appear, that the bad effects ascribed to cold applications in the above diseases, have arisen from the *indiscriminate* use of them in such cases as the above, in which neither the *temperature* nor the *sensation* of the patients admitted of their employment; and consequently in which, instead of promoting, they interrupted the actions going on at the time.

With regard to inflammation affecting internal parts, any attempt to ascertain the relation between the plastic actions and the pain in them must be entirely fruitless, if we are not permitted to reason from analogy, or from observations in extreme cases.

It is allowed that inflammation may exist in internal parts, an extravasation of coagulable lymph take place, and new vessels shoot into it; yet the patient experience no pain‡.

---

* Medical Reports, Vol. 1, Ch. 7. † Vol. 2, Ch. 2.

‡ See Hunter on Inflammation, p. 287. See also Baillie's Morbid Anatomy, 3d Edition, p. 59.

Inflammation

Inflammation of the heart and pericardium with adhesions*; of the pleura and lungs with adhesions†; of the stomach‡; of the peritoneum, &c. with adhesions||; of the kidneys§; have been found upon dissection after death to have taken place, although the patients had complained of no pain (certainly not acute pain) in the part in which the inflammatory actions had been going on. And in numberless instances to which it cannot be necessary to refer, the degree of pain felt has been incomparably less when the action was considerable, than in other cases in which few or no marks of inflammatory *action* could be detected. From these facts, is it not allowable to infer that the same principle prevails in internal as in external inflammation?—and that in both, the pain indicates that the *action* is not equal to the *efforts* to act? If this is granted, we must conclude that the affusions, adhesions, suppurations, &c. found in some fatal cases of inflammation attended with violent pain, took place mostly or entirely after the pain abated, which it always does, if I am not mistaken, some considerable time before the fatal termination of the disease.

### 3. OF THE DILATATION OF THE BLOOD VESSELS IN PARTS INFLAMED.

If we reflect upon the nature of the plastic actions; that the organization is to be repaired by them, or new vessels formed; and that the materials for these actions are to be derived from the blood; it cannot but appear that the slow state of the circulation and increased quantity of blood which constitute so constant a feature of inflammation, are well adapted if not essentially necessary to the undisturbed performance of these actions. For as a very rapid motion of the food intended for nourishment through the primæ viæ would be totally incompatible with its digestion and absorption; so, an increased velocity of the blood through the vessels of a part in which the actions of formation and repair are to be accomplished, instead of being favourable to increased plastic action would, it

* Burns on the Diseases of the Heart. Med. and Phys. Journ. Vol. 25, p. 396.

† Morgagni de Sed. et Caus. Ep. 21. n. 9. also n. 17. Ep. 3. n. 20—n. 26. Edin. Med. and Surg. Journ. Vol. 6, p. 437.

‡ Ibid. Vol. 7, p. 160.

|| Morgagni de Sed. et Caus. Ep. 17. n. 17. Ep. 53. n. 3. Edin. Med. and Surg. Journ. Vol. 4, p. 187; also Vol. 2, p. 409.

§ Van Swieten Comment. in Boerh, § 994.

it is self-evident, prevent that due separation of the materials without which these actions cannot be performed. But numerous facts concur in shewing this to be a general law of the animal economy, that in parts, in which actions either of repair or formation, morbid or salutary, are to be performed, there a lasting or temporary provision for diminishing the velocity of the blood, and for causing it to accumulate in larger quantity is formed. The following facts seem to testify that this law prevails in health.

1st. Organs which are liable to great or continual waste from the exercise of their several functions, mental, animal, or vital, and which in consequence require frequent or continual actions of repair, have the blood vessels naturally so disposed as to render the circulation slow, as well as the quantity of blood abundant in them—as the brain, heart, and voluntary muscles.

2d. Similar provision for diminishing the velocity and increasing the quantity of blood, is found in organs whose function it is to separate from the blood the materials of the various secretions—as the liver, kidneys, &c.

3d. In infancy, in which the rapid growth of the body proves that the plastic actions are predominant, the motion of the blood in the extreme vessels appears to be slower, and the quantity, cæteris paribus, greater than in adults, as the flushed or mottled red and white appearance of the skin in infancy evinces: and every matron knows that children in whom the skin is pale and bloodless neither thrive so well nor grow so fast, as those in whom it exhibits this mottled appearance.

4th. During pregnancy a very remarkable increase of plastic action in the uterus occurs; and a no less remarkable change takes place in the size of its blood vessels, and in the quantity of blood accumulated in them. The arteries and veins of the uterus in its unimpregnated state, though numerous, are small. But after impregnation they undergo an astonishingly rapid enlargement. "Even before the ovum enters the uterus, we find the uterine artery as large as the barrel of a goose quill, and sending large branches round the cervex uteri and up the sides of the womb. As pregnancy advances, the trunks, but especially the branches, become still larger, particularly near the implantation of the placenta. The veins are enlarged in the same proportion as the arteries *" "The enlargement of the veins during pregnancy is such that the orifices of some of them, when divided, will

* Burn's Principles of Midwifery, B. 1, Ch. 15, Sect. 5.

admit admit even the end of a small finger *." That this vast enlargement of the arteries and veins must be attended with the effect of rendering the velocity of the blood almost insensible, as well as of increasing its quantity in the uterine vessels, is sufficiently obvious.

5th. In sleep a considerable increase of plastic action appears to take place in every part of the system, especially in the organs adapted to the mental and animal functions, to repair the waste the organization necessarily suffers in the waking state. This seems to be universally taken for granted. But during sleep the motion of the blood becomes slower than when the person is awake. This appears from the slowness of the pulse in sleep. I am aware that a very different cause has been assigned for this slow state of the pulse. But since this slowness of the pulse is spontaneous, since it answers so good a purpose, and since *mechanical compression of the brain* or *distention of its blood vessels*, would defeat that purpose, I hope it will appear to the intelligent reader that this slow state of the pulse (in healthy sleep at least) is more likely owing to this law of the animal economy, than to any mechanical cause.

These instances may suffice to shew the prevalence of this law in the healthy state of the body. It appears to be no less general in disease.

1. In the medullary sarcoma or fungus hæmatodes, not only the medullary mass is plentifully supplied with blood by its own vessels, but the vessels in the neighbourhood are greatly enlarged; a circumstance that always must render the motion of the blood in them slower than natural.

2. When the skin covering tumours is made to recede spontaneously (not mechanically stretched and thinned) as the tumour enlarges, the veins of the skin appear universally to become enlarged and varicose. In this enlarged state of the veins the velocity of the blood must be diminished. The veins of the skin covering the fungus hæmatodes, cancerous tumours, some cases of abscess, fatty tumours, &c. always exhibit this varicose appearance, when the skin recedes before the tumour by an apposition of new matter. When it is forcibly stretched and thinned the veins do not assume that appearance.

3. I have seen some cases in which the mamma preparatory to lactation have become enormously enlarged. An

* Denman's Introduction to the Practice of Midwifery, Ch. 3, Sect 1.

enlarged enlarged and varicose state of the veins preceded and accompanied this enlargement.

4. Universally this state of the circulation appears to exist, with certain modifications, in inflammation. Wherever plastic action is to be performed, whether it be slight or considerable, there an accumulation and slower motion of the blood is produced or attempted to be produced. Even in clean cuts, which heal without pain, the blood accumulates around the edges of the divided skin: and the slightest wound though confined to a point, becomes surrounded with a blush, (this is particularly visible in infants), which does not go entirely off till either the part be healed, or the materials thrown out for healing it.

Since therefore a very slow motion and large quantity of blood appear to be essentially necessary in parts where increased actions of formation or repair are to be performed, it seems highly probable that in parts, in which the quantity of blood is naturally too small, and the velocity with which it moves through the vessels naturally too great for the occasion—the first action of vessels when inflammatory action is excited there, will be, as Mr. Hunter supposed, an *action of dilatation;* than which, it is probable, nothing can be so well adapted to produce that slow state of the circulation which is necessary to the easy performance of the intended action.

But it is a remarkable circumstance, that parts in which the velocity of the blood is naturally greatest, and the quantity inconsiderable, are always most painful under inflammation. This is the case with all the dense colourless membranes, such as the tendons, ligaments, pleura, peritoneum, dura mater, &c. in which the quantity of red blood in their natural state is so small and its velocity so great, that little or no sensible redness is produced by it. The small vessels of these membranes, therefore, must be supposed to have most occasion for an exertion of this action of dilatation when extraordinary plastic action is to be performed in them. But it is obvious that if they stand most in need of dilatation, they also must in general present most resistance to its being accomplished. And this may be one reason why inflammation is so much more painful when it affects these dense colourless membranes, than when it takes place in parts more abundantly supplied with blood, and which more readily admit of dilatation, if that be necessary; as the substance of the lungs, liver, &c.

The action of dilatation in the vessels of inflamed parts does not appear to be a mere stretching of their coats—it is really a plastic action, the vessels becoming enlarged in capacity by an apposition of new matter, and sometimes also in thickness.

But

But since it has been shown that the blood ought to be at rest, or almost so, in order to the perfect accomplishment of this action, it is obvious that whatever increases the velocity of the blood must disturb the actions attempted, and more or less hinder them from being performed. Accordingly we find that when the velocity of the blood thrown from the heart remains unbroken till it reach an inflamed part, pain is uniformly produced at each diastole of the arteries. This appears to be the chief cause of the greater pain attending inflammation in dense colourless membranes.

By this unbroken impetus of the blood into inflamed parts, therefore, the actions to be performed appear to be interrupted, even after a sufficient quantity of blood is collected in the small vessels for the occasion. This is well illustrated by the sixth experiment related at the beginning of this paper, when treating of the state of the circulation in inflamed parts. The inflammation in that case arose from a burn. The parts were very red; and therefore it might be supposed the vessels already contained blood enough to furnish materials for the actions of repair, if they were not prevented by some other cause. Compression of the humeral artery allowed the blood to rest in the inflamed parts; the throbbing pain ceased; and the actions of repair appear to have been nearly completed in the course of three minutes afterwards. Yet the inflammation was appearing to increase before the artery was compressed.

The effect which resistance to the action of dilatation has of inducing pain independent of any other appretiable cause, is sometimes strikingly exemplified in utero-gestation. Towards the latter end of pregnancy it is very common for women to be affected with what are termed *spurious pains*. These pains are often very severe: but on examination no contraction of the uterus, or dilatation of its mouth are found to have taken place. Commonly in these circumstances I have found the membranes and head of the child high up, and the cervix uteri not completely dilated and somewhat rigid. By attending strictly to the effect of these pains I have found, that after a considerable interval of time, a very sensible dilatation of the cervix uteri had taken place; which must have been effected entirely by an action of dilatation or relaxation: as not the least symptom of contraction of the body of the uterus was distinguishable. But that the pain in such instances arises from resistance to the action of dilatation appears probable from this circumstance, that after venesection, or the operation of a laxative, the pain commonly has ceased; and the cervix uteri, notwithstanding, has been found more dilated in a given time than when the pain was urgent; still without contraction of the uterus or pressing forwards of its contents. When this spontaneous dilatation of the cervix or os uteri is performed with great rapidity, but without difficulty, no pain is felt. The only symptoms by which it may be suspected are trembling of the limbs or of the whole body; sickness, with or without vomiting; and sometimes an indescribable anxiety not referred to any particular part of the body. The following case appears to be an instance of dilatation, first of the cervix and then of the os uteri, without resistance. I was sent for in great haste to attend a woman who was said to be taken in labour. She was the mother of a large family, and commonly bore her children with little pain, and without *professional aid*. I went immediately. She said she had not had any pain, but that she was sure her labour had begun, because her reckoning was out, and she was seized with a shaking which was always the principal symptom in her labours. On examination I found the os tincæ still close, the head of the child so high as scarcely to be felt, and the cervix uteri oblong and undilated. During the two hours that I sat with her, she was twice seized with a violent shaking of the whole body, which lasted for about a minute each time, and then left her. The change that took place in the cervix uteri during these *two minutes* was remarkable. At the end of the second shaking fit the cervix was quite obliterated, and the head of the child came to rest upon the os uteri, which, however, remained close. Yet during the time of the shakings, I could perceive no tension as if the contents of the uterus were pressed forwards; nor by applying the hand to the abdomen was the least symptom of contraction of the body of the uterus discernable. She now rested for a month. At the expiration of that time I was again sent for, but the child was born before I arrived. She had been several times seized with the shaking fit, such as I saw her have, at the interval of a few minutes between each, but denied she had felt *pain*. She knew she said that the child was advancing, but the sensation it produced could not be called pain. This is not a common case. But there are few labours in which opportunities are not given of observing the obvious effects of this action of dilatation when it comes to be little resisted, in diminishining the pain and facilitating the progress of the labour.

I have now examined all the *vital* actions which have been supposed, or which can be easily conceived to occur in inflammation: and if the observations of others agree with the above, it will be granted that the *pain* in inflamed parts is inseparably connected with interruption of the plastic actions; and therefore, that it is a further confirmation of the

Principle

Principle of Sensation. But even although the above observations are allowed on the whole to be correct, still one question respecting the cause of the sensation in inflammation suggests itself, viz. May not the pain depend not so much on the interruption of the action in the part singly, as on that action, being interrupted, giving an impression to the nerves, which is conveyed through them to the sensorium? Some facts appear to countenance this idea. The subject, however, is avowedly obscure. The word *impression* refers only to the agent, not to the manner the nerves are affected by it. Perhaps it might not be impossible to prove that *impressions* communicate sensation only as they interrupt action in the nerves; and that when vital action in the nerves ceases, impressions fail to communicate sensation. But I decline this subject at present, which might lead to discussions apparently more metaphysical than useful; satisfied with having shown that the pain in inflammation is caused by interrupted action, and that *actions* strictly morbid in their nature and fatal in their consequences may exist, without *pain* and without consciousness: facts, which I presume to hope, may be found neither void of interest nor utility.

Postscript.—In these papers on Sensation, I have had frequent occasion to employ the term *vital power*. I have not attempted to define that term, as it did not appear to me that I used it in a sense essentially different from that which is commonly attached to it. Generally, if not universally, it is acknowledged that actions or functions are performed on the living body, not explicable on any purely chemical or mechanical priciples *hitherto known*. The common sense of mankind revolts against the idea that these functions are affected by accident or without an established cause. They have, therefore, agreed in ascribing the phenomena of life to the influence of an internal principle, *sui generis*, to which the name of *Vital Power* has been given. In taking for granted the existence of such a power we no more offend against the laws of true philosophy, than in ascribing the phenomena of gravitation to a power inherent in matter. As it is by the *action* of the power of gravity that the planets are retained in their orbits; so it appears that the action of the vital power is that which gives form and motion to the materials of which the body is composed. The existence of either power cannot be proved but by its effects. But on attentively considering these effects, the understanding, if I am not mistaken, is as strongly impressed with a persuasion of the existence of the one, as of the other. I have elsewhere said, "it is not difficult to conceive that an active intelligent power should *feel* when its motions are interrupted." There is an

 evident evident inaccuracy in this expression, which I conceive has given rise to the observations of your correspondent Mr. Woodham. The sentence, to convey my meaning, should have run thus: "it is not difficult to conceive that an active intelligent power should feel when its *actions* are interrupted." The word motions refers properly to the effect of the vital actions upon matter; and the expression as it formerly stood implied an hypothesis which it was certainly far from my intention to assume. I therefore feel myself highly obliged to Mr. Woodham for having called my attention to that expression, and furnishing me with an opportunity to correct the inaccuracy. But if sensation uniformly depends on interrupted vital action; and if, as Mr. Woodham asserts, "intelligence is mind and sensation a mental affection," I am yet to learn wherein I have deviated from the strictest principles of philosophy, either in the above expression as it now stands corrected, or in concluding from *facts* that the vital power feels when its actions are interrupted or obstructed.

T. SMITH.

*Bristol,* 1811.

